# First-Line ICI Monotherapies for Advanced Non-small-cell Lung Cancer Patients With PD-L1 of at Least 50%: A Cost-Effectiveness Analysis

**DOI:** 10.3389/fphar.2021.788569

**Published:** 2021-12-21

**Authors:** Qiao Liu, Zhen Zhou, Xia Luo, Lidan Yi, Liubao Peng, Xiaomin Wan, Chongqing Tan, Xiaohui Zeng

**Affiliations:** ^1^ Department of Pharmacy, The Second Xiangya Hospital of Central South University, Changsha, China; ^2^ Menzies Institute for Medical Research, University of Tasmania, Hobart, TAS, Australia; ^3^ Department of Nuclear Medicine/PET Image Center, The Second Xiangya Hospital of Central South University, Changsha, China

**Keywords:** non-small cell lung cancer, PD-L1 expression, cemiplimab, pembrolizumab, atezolizumab, cost-effectiveness

## Abstract

**Objective:** Three immune checkpoint inhibitors (ICIs), pembrolizumab, atezolizumab and cemiplimab, have been successively approved as first-line treatments for advanced non-small-cell lung cancer (NSCLC) patients with programmed cell death ligand 1(PD-L1) expression of at least 50%. This study was designed to compare the cost-effectiveness of these three novel therapies in this patient population.

**Material and Methods:** Using Markov model and network meta-analysis, we conducted separate cost-effectiveness analyses for cemiplimab, pembrolizumab and atezolizumab among advanced NSCLC patients with PD-L1 of at least 50% from the United States health care sector perspective. Health states included progression-free survival, progressive disease, end-stage disease, and death. Clinical efficacy and safety data were derived from phase III clinical trials and health state utilities and costs data were collected from published resources. Two scenario analyses were conducted to assess the impact of varying subsequent anticancer therapies on the cost-effectiveness of these 3 ICIs and cost-effectiveness of pembrolizumab combined with chemotherapy versus these 3 first-line ICI monotherapies.

**Results:** In base case analysis, cemiplimab compared with pembrolizumab was associated with a gain of 0.44 quality-adjusted life-years (QALYs) and an increased cost of $23,084, resulting in an incremental cost-effectiveness ratio (ICER) of $52,998/QALY; cemiplimab compared with atezolizumab was associated with a gain of 0.13 QALYs and a decreased cost of $104,642, resulting in its dominance of atezolizumab. The first scenario analysis yielded similar results as our base case analysis. The second scenario analysis founded the ICERs for pembrolizumab plus chemotherapy were $393,359/QALY, $190,994/QALY and $33,230/QALY, respectively, compared with cemiplimab, pembrolizumab and atezolizumab.

**Conclusion:** For advanced NSCLC patients with PD-L1 of at least 50%, cemiplimab was a cost-effective option compared with pembrolizumab and a dominant alternative against atezolizumab. Our scenario analysis results supported the cemiplimab plus chemotherapy as a second-line therapy and suggested an extended QALY but overwhelming cost linking to pembrolizumab plus chemotherapy.

## Introduction

Lung cancer is the most common malignancy and the leading cause of cancer mortality worldwide (William et al., 2009). Non-small cell lung cancer (NSCLC) represents approximately 85% of all lung cancers, and up to 46% of NSCLC cases have advanced diseases at the time of diagnosis (Miller et al., 2020). Decision making on the standard first-line treatment for advanced NSCLC is personalized, based mainly on driver aberration types and programmed cell death ligand 1 (PD-L1) expression levels (Ettinger et al., 2021). Over the past few decades, treating NSCLC patients with traditional platinum-doublet chemotherapy has obtained unsatisfactory therapeutic effect, with a median overall survival (OS) of less than 1 year and a 5-year survival rate of nearly 18% (William et al., 2009). Accumulating evidence have reported that a higher expression of PD-L1 was associated with a poorer clinical prognosis and greater resistance to chemotherapy in NSCLC patients (Creelan, 2014). Immune checkpoint inhibitors (ICIs), as a novel class of anticancer drugs, have therefore hold a great therapeutic potential on the management of advanced NSCLC patients, especially those with a high level of PD-L1 expression (Gridelli and Casaluce, 2018; Hanna et al., 2020).

Up to now, the United States Food and Drug Administration (FDA) has successively approved 3 ICI monotherapies for the first-line treatment of advanced NSCLC patients with at least 50% tumor cells expressing PD-L1 (US Food and Drug Administration, 2016; US Food and Drug Administration, 2021a; US Food and Drug Administration, 2021b). Pembrolizumab is the first approved ICI that has demonstrated significantly greater survival benefits and fewer adverse events (AEs) compared with platinum-based chemotherapy in the clinical trials of KEYNOTE-024 and KEYNOTE-042 (Reck et al., 2016; Mok et al., 2019). In May 2020, atezolizumab became the second approved ICI proven effective among PD-1 selected advanced NSCLC patients based on the IMpower110 trial (Herbst et al., 2020). More recently in February 2021, data from the EMPOWER-Lung 1 clinical trial, documented a significantly improved OS and progression-free survival (PFS) with cemiplimab in patients with advanced NSCLC with PD-L1 of at least 50%, when compared with chemotherapy (Sezer et al., 2021). Informed by the clinical evidence, cemiplimab was approved as a new first-line option for this patient population.

It was estimated that there were 116,700 patients in the United States (United States) developing advanced NSCLC in 2020 (American Cancer Society., 2021), and nearly 25–35% of them are expected to express PD-L1 in at least 50% of tumor cells (D'Incecco et al., 2015; Kerr et al., 2015). This means that about 40,800 patients are potentially eligible for ICI therapies. Given the huge population of beneficiaries and the expected negative financial consequences, comparing the cost-effectiveness of these ICIs among this patient population in the United States is necessary to determine their appropriateness for widespread use (Tsevat and Moriates, 2018). Several previous US-based studies have evaluated the cost-effectiveness of pembrolizumab or atezolizumab versus platinum-based chemotherapy in the first-line setting of advanced NSCLC patients with PD-L1 of at least 50% (Huang et al., 2017; Peng et al., 2021). However, the generalizability of their findings to real-world settings may be limited, in which the ICIs are typically used preferentially over traditional chemotherapy. The priority of these 3 first-line ICI monotherapies has yet to be determined.

To inform the resource allocation decision, we conducted this study to compare the cost-effectiveness of cemiplimab with pembrolizumab and atezolizumab as the first-line treatment for advanced NSCLC patients with PD-L1 of at least 50% from the United States health care sector perspective.

## Materials and Methods

### Overview

Through mathematical modeling using TreeAge Pro software (version 2021, https://www.treeage.com/) and network meta-analysis (NMA) implemented in R software (version 4.0.4, http://www.r-project.org), we conducted an indirect cost-effectiveness comparison of 3 first-line ICI monotherapies for advanced NSCLC patients with PD-L1 of at least 50% from the United States health care sector perspective. This study collected and studied existing data, including clinical efficacy and safe data from published Phase III clinical trials, health state utilities and costs data from previous literature and publicly available United States database. Therefore, it is exempt from ethic review. Our study follows the Consolidated Health Economic Evaluation Reporting Standards (CHEERS) reporting guideline.

### Patients and Treatment

Three potential competing first-line ICI monotherapies were assessed in the model: cemiplimab, pembrolizumab and atezolizumab. A hypothetical cohort of advanced NSCLC patients aged 18 years or older with PD-L1 expressed in at least 50% of tumor cells and without driver molecular alterations was created in our model. We did not incorporate a platinum-based chemotherapy arm into the model although it is a common comparator in clinical trials, because it is no longer recommended as a preferred first-line treatment in the latest National Comprehensive Cancer Network (NCCN) guidelines for this patient population (Ettinger et al., 2021).

After progressed on first-line ICI monotherapies, the subsequent anticancer therapies were provided if there were sustained survival benefits. Patients assigned to cemiplimab had the option to continue cemiplimab with the addition of 4 cycles of chemotherapy (Sezer et al., 2021); patients assigned to pembrolizumab and atezolizumab would be permitted to receive chemotherapy, immunotherapy, and targeted therapy (Reck et al., 2016; Mok et al., 2019; Herbst et al., 2020). The usage of subsequent anticancer drug was based on NCCN guidelines as well as the availability of clinical data (Reck et al., 2016; Mok et al., 2019; Herbst et al., 2020; Ettinger et al., 2021). Supplementary Table S1 provided detailed information on first-line and subsequent treatment regimens.

### Model Construction

**FIGURE 1:**
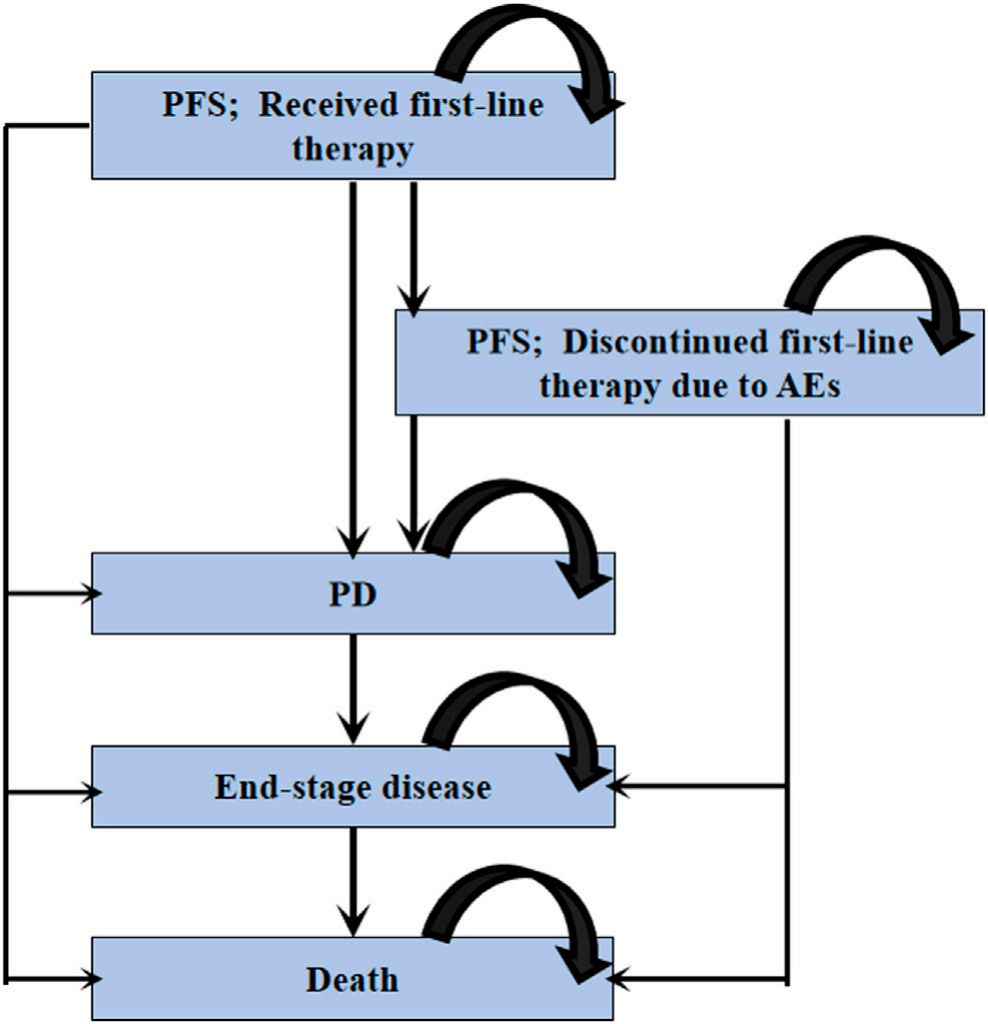
Diagram of Markov model.

We constructed a Markov model consisting of four health states: PFS, progressive disease (PD), end-stage disease and death (Figure 1). All patients initially entered the PFS health state, then received first-line cemiplimab, pembrolizumab or atezolizumab monotherapy until disease progression or intolerable toxicity. Individuals who experienced disease progression during first-line treatment could move to the PD health state and receive subsequent anticancer therapies. Individuals who were not eligible for subsequent anticancer therapies finally entered into end-stage health state and proceeded to best supportive care (BSC) (Ettinger et al., 2021). To reflect the actual clinical practice, patients were assumed to receive palliative care before death.

We built the Markov model with a 3-week cycle length to project the health and economic outcomes associated with each treatment over a lifetime horizon. The main output of the model was the incremental cost-effectiveness ratio (ICER) between the compared treatment strategies, which was calculated as the cost for each additional quality-adjusted life-year (QALY) gained. Costs were reported in 2021 United States dollars and an annual discount of 3% was adopted for both cost and health outcomes. This analysis chose a willingness-to-pay (WTP) threshold of $100,000 per QALY as a cost-effectiveness measure of one regimen relative to another alternative regimen (Neumann et al., 2014).

### Survival and Health State Utilities

For first-line cemiplimab, transition probabilities were calculated from the EMPOWER-Lung 1 trial. Briefly, the OS and PFS data were graphically extracted from the published Kaplan-Meier curves, then fitted and extrapolated by log-logistic survival distribution based on statistical measures of goodness-of-fit [Akaike information criterion (AIC) and Bayesian information criterion (BIC)] (Supplementary Table S2 and Figure 1). The final the log-logistic theta (*θ*) and kappa (*κ*) parameters were computed by R software. The survival probability at a given time cycle t was calculated following this formula: $\;S\left(t\right)=1/\left[1+exp\left(\theta \right)t^{\text{κ}}\right]$ . For first-line pembrolizumab and atezolizumab, the estimation of transition probabilities were based on the hazard ratios (HRs) of PFS and OS for the two alternative strategies relative to cemiplimab, which was used to adjust survival probability ( $S{\left(t\right)}_{alternative\;strategies}=[1/{\left[1+exp\left(\theta \right)t^{\kappa }\right]}^{HRs}$ ) (Wu et al., 2012). Given the absence of relevant clinical trials with head-to-head comparisons, the HRs were generated in a NMA implemented in the R software, with using data derived from published clinical trials that compared ICI monotherapy versus chemotherapy in the target population, including EMPOWER-Lung 1, KEYNOTE-024, KEYNOTE-042 and IMpower110 trials (Supplementary Table S3 **)**. All log-logistic parameters and HRs used in the model were presented in Table 1.

**TABLE 1:** Model inputs.

Parameters	Baseline value	Ranges	Distribution	Source
Survival				
Log-logistic survival model for first-line cemiplimab				
OS	*θ*=0.02432, *κ*=1.11748	-	-	Estimated^a^
PFS	*θ*=0.09810, *κ*=1.14350	-	-	Estimated^a^
HRs for first-line pembrolizumab vs cemiplimab				
OS	1.17	0.82–1.27	LogNormal	Estimated^b^
PFS	1.21	1.12–1.48	LogNormal	Estimated^b^
HRs for first-line atezolizumab vs cemiplimab				
OS	1.04	0.63–1.71	LogNormal	Estimated^b^
PFS	1.16	0.32–4.32	LogNormal	Estimated^b^
1-Cycle probability of treatment discontinuation due to AEs				
First-line cemiplimab	0.00303	0.00151–0.00454	Beta	Estimated^c^
First-line pembrolizumab	0.00499	0.00250–0.00749	Beta	Estimated^c^
First-line atezolizumab	0.00188	0.00094–0.00283	Beta	Estimated^c^
Costs				
Cemiplimab price/mg	27.58	13.79–41.37	Gamma	Centers for Medicare and (2021a)
Pembrolizumab price/mg	51.35	25.67–77.02	Gamma	Centers for Medicare and (2021a)
Atezolizumab price/mg	7.98	3.99–11.97	Gamma	Centers for Medicare and (2021a)
Ramucirumab price/mg	12.48	6.24–18.71	Gamma	Centers for Medicare and (2021a)
Nivolumab price/mg	28.90	14.45–43.34	Gamma	Centers for Medicare and (2021a)
Docetaxel price/mg	0.54	0.27–0.81	Gamma	Centers for Medicare and (2021a)
Pemetrexed price/mg	7.42	3.71–11.13	Gamma	Centers for Medicare and (2021a)
Gemcitabine price/mg	0.02	0.01–0.03	Gamma	Centers for Medicare and (2021a)
Paclitaxel price/mg	0.13	0.07–0.20	Gamma	Centers for Medicare and (2021a)
Carboplatin price/mg	0.05	0.02–0.07	Gamma	Centers for Medicare and (2021a)
Cisplatin price/mg	0.18	0.09–0.28	Gamma	Centers for Medicare and (2021a)
Advent event (first-line cemiplimab)	351.05	175.52–526.57	Gamma	Agency for Healthcare Res, (2021)
Advent event (first-line pembrolizumab)	1,092.31	546.16–1,638.47	Gamma	Agency for Healthcare Res, (2021)
Advent event (first-line atezolizumab)	713.04	356.52–1,069.56	Gamma	Agency for Healthcare Res, (2021)
Administration intravenous, first hour	148.30	74.15–222.45	Gamma	Centers for Medicare and (2021b)
Administration intravenous, additional hour	31.40	15.70–47.10	Gamma	Centers for Medicare and (2021b)
Monthly physician visit	183.19	91.60–274.79	Gamma	Centers for Medicare and (2021b)
Three-monthly imaging	117.59	58.80–176.39	Gamma	Centers for Medicare and (2021b)
Monthly supportive care	637.00	318.50–955.50	Gamma	Criss et al. (2019)
Death associated costs	9,433.00	4,716.50–14149.50	Gamma	Criss et al. (2019)
Utilities				
≥12 months prior to death	0.805	0.767–0.843	Beta	Brahmer et al. (2017), Hatswell et al. (2014)
6-12 months prior to death	0.726	0.684–0.767	Beta	Brahmer et al. (2017), Hatswell et al. (2014)
1-6 months prior to death	0.632	0.592–0.672	Beta	Brahmer et al. (2017), Hatswell et al. (2014)
≤1 months prior to death	0.573	0.425–0.650	Beta	Brahmer et al. (2017), Hatswell et al. (2014)
Disutility for first-line cemiplimab	0.006	0.003–0.009	Beta	Estimated^d^
Disutility for first-line pembrolizumab	0.014	0.007–0.020	Beta	Estimated^d^
Disutility for first-line atezolizumab	0.005	0.003–0.008	Beta	Estimated^d^
Others				
Body weight (kg)	70.32	69.71–70.93	Normal	Criss et al. (2019)
Body surface area (meters^2^)	1.79	1.78–1.80	Normal	Criss et al. (2019)
Creatinine clearance rate(ml/min)	70.00	35.00–105.00	Normal	Zhang et al. (2020)

a The log-logistic distribution parameters, theta (*θ*) and kappa (*γ*) were estimated based on survival data reported in the EMPOWER-Lung 1 trial

b The HRs were generated using network meta-analyses

c Estimated in the Supplementary Table S3

d Estimated in the Supplementary Table S5

OS, overall survival; PFS, progression-free survival; HRs, hazard ratios; AEs, adverse events.

We also incorporated in the model the discontinuation of first-line ICI monotherapy owing to adverse events (AEs), with transition probabilities estimated from clinical trials (Reck et al., 2016; Mok et al., 2019; Herbst et al., 2020; Sezer et al., 2021). The following formula was applied to convert the probabilities of AEs-related treatment discontinuation during a clinical trial period into a 1-cylce probability of the events: p=1−exp(−rt) , where p indicates the probability, r is the instantaneous rate and t is the time period (Supplementary Table S4) (Briggs and Claxton, 2006). Finally, the long-term observed survival data for advanced NSCLC patients from the Surveillance, Epidemiology, and End Results data from 2000 to 2018 were applied to estimate survival after patients entering end-stage disease health state, to ensure the OS of advanced NSCLC closely reflect the real-world performance (Supplementary Table S5) (National Cancer Institute Surveillance and End Results Program, 2021).

For all model groups, the health utilities were derived from the European Quality of Life 5 Dimensions-3 Level (EQ-5D-3L) data reported in the KEYNOTE-024 trial (Brahmer et al., 2017). The time-to-death approach was applied to reflect the decline in quality-of-life in patients with advanced NSCLC as they approach death (Hatswell et al., 2014). In addition, the utility decrements for common grade III/IV AEs as a result of first-line treatment were considered in our model (Supplementary Table S6) (Nafees et al., 2017).

### Cost Estimates

We collected direct medical costs from the United States health care sector perspective, including first- and second-line drug acquisition and administration costs, AEs management costs and general treatment costs of advanced NSCLC (such as routine follow-up costs, BSC costs, and death-associated costs). Cost inputs used in the model were outlined in Table 1.

Drug prices were collected from the Centers for Medicare and Medicaid Services (CMS) 2021 Average Sales Price drug Pricing Files (Centers for Medicare and, 2021a). In calculating the drug dosage, we used a body weight of 70.32 kg, a body surface area of 1.79 m^2^ and a creatinine clearance rate of 70 ml/min for model base case patients (Criss et al., 2019; Zhang et al., 2020). Drug administration costs were searched from the CMS Physician Fee Schedule Look-up Tool updated in January 2021 (Centers for Medicare and, 2021b). For drugs with infusion time requirements, we modeled the duration of ICI monotherapy and chemotherapy as 1 h per cycle and 3 h per cycle, respectively (Zhang et al., 2020).

Costs for managing grade III/IV AEs with an incidence of at least 1% were considered in the model (Reck et al., 2016; Mok et al., 2019; Herbst et al., 2020; Sezer et al., 2021). To calculate the AEs costs for each first-line treatments, we multiplied the incidence of each AE observed in the corresponding clinical trials by its management cost, and then summarize these costs to generate the cumulative cost. The AEs management costs were derived from the Healthcare Cost and Utilization Project (HCUP) using Clinical Classification Software Refined (CCSR) diagnosis (Supplementary Table S6) (Agency for Healthcare Res, 2021). We assumed that patients would receive a monthly physician visit and a three-monthly imaging examination during the routine follow-up. BSC cost and death-associated costs were sourced from published literature (Criss et al., 2019).

### Sensitivity Analysis

To assess the uncertainty in the model, both deterministic sensitivity analyses (DSA) and probabilistic sensitivity analyses (PSA) were employed. During DSA, model parameters varied individually, while other parameters were fixed to determine their roles in the ICERs. We defined the reported 95% (confidence Intervals) CIs as the test ranges for HRs and Utility values, whereas the ±50% of the baseline values as the test ranges for other parameters. During PSA, each model parameter followed an appropriate statistical distribution, and 10,000 Monte Carlo simulations were performed using random sampling of model parameters from the distributions each time. All ranges and distributions of model parameters were detailed in Table 1.

We also conducted two scenario analyses. First, we assumed that the same subsequent anticancer therapy regimen (cemiplimab plus 4 cycles chemotherapy) was used in these three first-line ICI monotherapy groups, using survival data from the EMPOWER-Lung 1 trial. This scenario allowed a brief comparative analysis of different subsequent anticancer therapies from the perspectives of cost and effectiveness. In the second scenario analysis, we incorporated a pembrolizumab plus chemotherapy group in our model and used the results of a MNA focusing on the efficacy of first-line pembrolizumab versus pembrolizumab plus chemotherapy in the treatment of advanced NSCLC (Kim et al., 2019). This scenario allowed us to conservatively predict the cost-effectiveness of ICI combined with chemotherapy versus ICI monotherapy in the absence of head-to-head clinical data.

## Results

### Incremental Cost-Effectiveness Ratios

In our base case analysis, treating patients with first-line cemiplimab monotherapy compared with first-line pembrolizumab and atezolizumab monotherapy were associated with improved survivals of 0.44 QALYs and 0.13 QALYs, respectively. In addition, the healthcare cost caused by cemiplimab was greater than pembrolizumab ($231,338 vs. $217,456) but substantially lower than atezolizumab ($231,338 vs $332,126) (Table 2). The results showed that first-line cemiplimab was a cost-effective option compared with first-line pembrolizumab (ICER = $23,083/QALY), and a dominant alternative against first-line atezolizumab when the WTP threshold set as $100,000/QALY.

**TABLE 2:** Summary of simulation results

Analysis	Cost, $	QALYs	Incremental		ICER, $/QALY
			Cost, $	QALYs	
Base case analysis					
First-line cemiplimab	231,338	3.10	NA	NA	
vs. First-line atezolizumab	335,980	2.97	−104,642	0.13	Dominated
vs. First-line pembrolizumab	208,254	2.65	23,084	0.44	52,998 (cost-effective)
First scenario analysis					
First-line cemiplimab	231,338	3.10	NA	NA	
vs. first-line atezolizumab	302,274	3.02	−70,937	0.08	Dominated
vs. first-line pembrolizumab	219,623	2.85	11,714	0.25	47,124 (cost-effective)
Second scenario analysis					
First-line Pembrolizumab plus Chemotherapy	350,281	3.40	NA	NA	
vs. First-line atezolizumab	335,980	2.97	14,301	0.43	33,230 (cost-effective)
vs. First-line pembrolizumab	208,254	2.65	142,027	0.75	190,994 (not cost-effective)
vs. First-line cemiplimab	231,338	3.10	118,943	0.30	393,359 (not cost-effective)

QALY, quality-adjusted life-year; ICER, incremental cost-effectiveness ratio.

In the first scenario analysis, the use of cemiplimab plus chemotherapy, as the only subsequent anticancer therapy, resulted in incremental effectiveness of 0.19 QALYs (2.85 vs. 2.65 QALYs) and 0.05 QALYs (3.02 vs. 2.97 QALYs) in the first-line pembrolizumab and atezolizumab groups, compared with our base case results. In the second scenario analysis, we incorporated a pembrolizumab plus chemotherapy group in our model and found that treating patients with pembrolizumab plus chemotherapy in the first-line setting was associated with a mean cost of $350,281 and a mean survival of 3.40 QALYs. The model results showed that, when compared with cemiplimab, pembrolizumab and atezolizumab, the ICERs for pembrolizumab plus chemotherapy were $393,359/QALY, $190,994/QALY and $33,230/QALY, respectively (Table 2).

### Sensitivity Analysis

**FIGURE 2:**
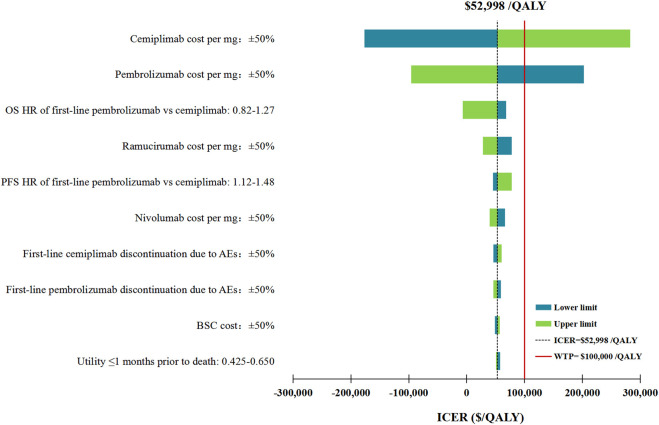
Deterministic sensitivity analysis for the base case analysis. ICER, incremental cost-effectiveness ratios; QALY, quality-adjusted life-years; OS, overall survival; PFS, progression-free survival; HR, hazard ratios; AEs, adverse events; BSC, best supportive care.

The DSA of the base case analysis revealed that, the fluctuation of any tested model parameter, except for the price per mg of cemiplimab and pembrolizumab, was unable to change the cost-effectiveness advantage of first-line cemiplimab over first-line pembrolizumab. More specifically, either increasing the price per mg of cemiplimab from $27.58 to more than $30.40 or decreasing the price per mg of pembrolizumab from $ 51.35 to less than $ 43.26, would bring the ICERs above the WTP threshold of $100,000/QALY. Other model parameters, such as the HRs of OS and PFS for the fist-line pembrolizumab strategy relative to the fist-line cemiplimab, and the price per mg of second-line ramucirumab had a moderate influence on the ICER. The top 10 parameters by magnitude of effect on the ICER were presented in Figure 2.

In the first scenario analysis, the most influential parameters with the ability to reverse our model results regarding the cost-effectiveness of first-line cemiplimab versus pembrolizumab remained the price per mg of cemiplimab and pembrolizumab. In addition, the price of subsequent anticancer therapy drugs, such as the price per mg of ramucirumab and nivolumab, which had considerable impacts on our base case analysis results, was no longer ranked in the top 10 parameters with the greatest associations with the ICER between first-line cemiplimab and pembrolizumab (Supplementary Figure S3). In the second scenario analysis, the ICER between first-line pembrolizumab plus chemotherapy and cemiplimab was most sensitive to the OS HRs, followed by the price per mg of cemiplimab and pembrolizumab. Other model parameters varied but did not change the preferred strategy assuming a WTP threshold of $100,000/QALY (Supplementary Figure S4).

**FIGURE 3:**
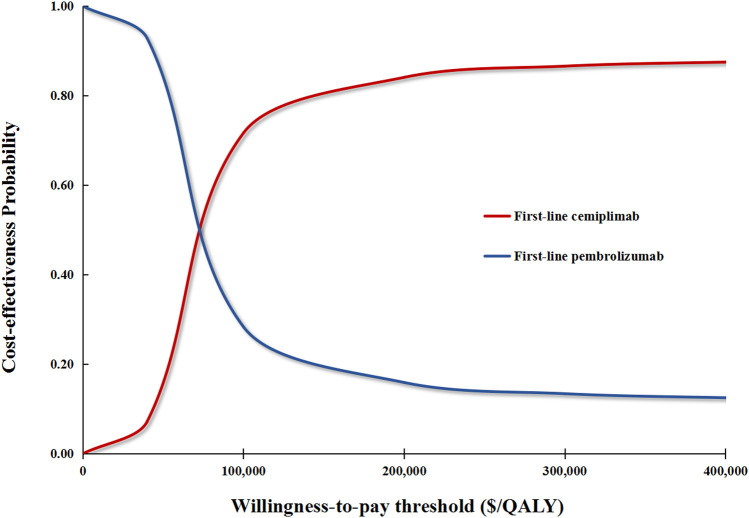
Cost-effectiveness acceptability curves for the base case analysis. The red curve signifies the probability of first-line cemiplimab being cost-effective against first-line pembrolizumab under different WTP thresholds. QALY, quality-adjusted life-year.

In performing PSA for the base case analysis, first-line cemiplimab was cost-effective in 71.1% of iterations and dominant in 11.2% of iterations compared with first-line pembrolizumab (Figure 3). In the first scenario analysis, first-line cemiplimab was cost-effective in 79.4% of iterations and dominant in 18.2% of iterations compared with first-line pembrolizumab. In the second scenario analysis, compared with first-line cemiplimab, first-line pembrolizumab plus chemotherapy was not cost-effective in 72.2% of iterations and was dominated in 32.1% of iterations.

## Discussion

In this cost-effectiveness analysis, our base case results suggested that cemiplimab was a cost-effective treatment strategy in comparison to pembrolizumab with an ICER of 52,998/QALY, and a dominant alternative against atezolizumab. In our first scenario analysis examining the impact of subsequent anticancer therapy regimen on the model outputs, we reached the same conclusion as the base case analysis. In the second scenario analysis evaluating the cost-effectiveness for different ICI treatment paradigms, model results suggested that first-line pembrolizumab plus chemotherapy was inferior to cemiplimab monotherapy and pembrolizumab monotherapy but was superior to atezolizumab monotherapy. To our best knowledge, this is the first cost-effectiveness analysis focusing on the newly approved ICI cemiplimab for advanced NSCLC with PD-L1 of at least 50% from the United States perspective and the first to compare these 3 ICI monotherapies (pembrolizumab, atezolizumab and cemiplimab) approved as the preferred upfront therapy. Given that more than one-tenth of the newly diagnosed lung cancer cases in the world occurred in the United States (Bray et al., 2018; Miller et al., 2020), our findings will have a significant impact on reducing both national and global lung cancer burden at a population-based level by providing the useful evidence on the comparative cost-effectiveness of three novel immunotherapies. The United States is a representative developed country which implies that our study findings may also be applicable to countries with similar health sectors.

Sensitivity analyses revealed that the price of ICIs plays a crucial role in determining the cost-effectiveness of one regimen relative to another alternative regimen. Our results were in line with the findings of several previous studies (Criss et al., 2019; Wan et al., 2019; Watson et al., 2020), stimulating debates on pricing scheme for cancer drugs. The United States policy researchers have paid great efforts on determining drug prices in terms of drug’s benefits, such as indication-specific pricing, value-based pricing and the “Netfiix Model” (Bach, 2014; Bach and Pearson, 2015; Trusheim et al., 2018). However, due to the relatively unrestrained pricing power given to drug manufacturers by the United States law, private insurers are unable to obtain reasonable drug prices (Prasad and Mailankody, 2016). As a result, drug prices in the United States are generally higher than those in other major industrialized countries (Comparative price report:, 2013), and are usually independent of drug novelty (Mailankody and Prasad, 2015). As cancer drug prices are already alarmingly high and rising faster than the prices of drugs used in other health care sectors in the United States (Bach, 2009; Mailankody and Prasad, 2014), there is an urgent need to update relevant policies to ensure the cancer drug prices commensurate with their clinical benefits. These price policy recommendations may include but not limited to value-based pricing as informed by this study (Bach and Pearson, 2015), price negotiation between governments and the pharmaceuticals (Tang et al., 2020), and government-subsidized medication schemes (Duckett, 2004). Apart from the drug price, HR is another equally important parameter that considerably influences the robustness of our model. It is worth noting that HR is an important factor in determining QALY gain, and it is relatively difficult to change it through policy intervention. Therefore, price adjustment would be the most realistic means that can be taken to make an ICI-based therapy cost-effective.

In the first scenario analysis, although our attempts to unify the subsequent anticancer therapy in these three first-line ICI monotherapy groups did not significantly change our results, the increases in QALYs in the first-line pembrolizumab and atezolizumab groups compared with our base case results may support a case for expanding the cemiplimab plus chemotherapy indication to second-line settings for advanced NSCLC patients with PD-L1 of at least 50%. The second scenario analysis results showed that first-line pembrolizumab plus chemotherapy was associated with incremental effectiveness compared with these 3 first-line ICI monotherapy, due mainly to a lower rate of early treatment failures with combination therapy than the ICI monotherapy (Di Federico et al., 2021).

This analysis has several notable strengths. First, we exhausted all available clinical trial data and authoritative MNA results to compare the lifetime healthcare cost and clinical outcomes of all first-line therapy preferentially approved by FDA, including three ICI monotherapies and one ICI combination therapy, which may add important cost-effectiveness evidence to inform the preferred treatment options for advanced NSCLC patients with PD-L1 of at least 50%. Second, the long-term survival estimates for model patients were based on the Surveillance, Epidemiology, and End Results data from 2000 to 2018. By using the real-world data, the underlying uncertainty caused by directly extrapolating survival from the fitted survival distributions was avoided (Wan et al., 2019). Third, we considered first-line treatment discontinuation due to AEs, as well as the impact of grade III/IV AEs on medical cost and utility in our model to refine the simulation of our model.

This study also has several limitations. First, due to the lack of clinical data comparing these 3 ICI monotherapies head-to-head, or ICI combined chemotherapy with ICI monotherapy with in one trial, the results of NMA were used for the analysis of an indirect cost-effectiveness comparison. Although the results from sensitivity analyses suggested that changing HRs did not alter the results of our base case analysis and the first scenario analysis, it may reverse the results of the second scenario analysis. Nonetheless, the second scenario analysis should be viewed as a tentative evaluation in the absence of head-to-head trials, and the model could be validated when more mature clinical data are available. Second, to simplify the model, we have made some assumptions regarding subsequent anticancer drugs, because the specific drugs information in corresponding clinical trials is not available. This assumption may bias the model against cost estimates. However, our findings were found to be robust over a wide range of variations in the price of subsequent anticancer drugs. Third, we modeled lower proportions of patients receiving second-line ICI in the first-line pembrolizumab and atezolizumab groups based on subsequent anticancer therapy data derived from clinical trials (Reck et al., 2016; Mok et al., 2019; Herbst et al., 2020; Sezer et al., 2021). There is an uncertainty regarding whether patients whose cancer progressed on first-line ICI monotherapy would continue to benefit from further ICI treatment. However, we explored this in our first scenario analysis by modeling the subsequent anticancer therapy of these 3 first-line ICI monotherapy as cemiplimab plus chemotherapy.

In conclusion, in this economic evaluation comparing the 3 approved first-line therapies for advanced NSCLC patients with PD-L1 of at least 50%, cemiplimab was a cost-effective treatment strategy compared to pembrolizumab, and a dominant alternative against atezolizumab. The results of our scenario analysis support the use of cemiplimab plus chemotherapy as a potential second-line therapy for this patient population and suggested that pembrolizumab plus chemotherapy was associated with extended QALY but an overwhelming cost. Our findings highlight the need for the United States policymakers to develop pricing schemes that can make drug prices commensurate with their values.

## Data Availability

The original contributions presented in the study are included in the article/Supplementary Material, further inquiries can be directed to the corresponding authors.

## References

[B1] Agency for Healthcare Research and Quality, U.S. Department of Health & Human Services. (2021). Healthcare Cost and Utilization Project. Availableat: https://hcupnet.ahrq.gov (Accessed May 15, 2021)

[B2] American Cancer Society (2021). Key Statistics for Lung Cancer. Availableat: https://www.cancer.org/cancer/lung-cancer.html (Accessed May 6, 2021)

[B3] Bach P. B. (2014). Indication-specific Pricing for Cancer Drugs. JAMA 312 (16), 1629–1630. 10.1001/jama.2014.13235 25279433

[B4] Bach P. B. (2009). Limits on Medicare's Ability to Control Rising Spending on Cancer Drugs. N. Engl. J. Med. 360 (6), 626–633. 10.1056/NEJMhpr0807774 19176475

[B5] Bach P. B. Pearson S. D. (2015). Payer and Policy Maker Steps to Support Value-Based Pricing for Drugs. JAMA 314 (23), 2503–2504. 10.1001/jama.2015.16843 26619354

[B6] Brahmer J. R. Rodríguez-Abreu D. Robinson A. G. Hui R. Csőszi T. Fülöp A. (2017). Health-related Quality-Of-Life Results for Pembrolizumab versus Chemotherapy in Advanced, PD-L1-Positive NSCLC (KEYNOTE-024): a Multicentre, International, Randomised, Open-Label Phase 3 Trial. Lancet Oncol. 18 (2), 1600–1609. 10.1016/S1470-2045(17)30690-3 29129441

[B7] Bray F. Ferlay J. Soerjomataram I. Siegel R. L. Torre L. A. Jemal A. (2018). Global Cancer Statistics 2018: GLOBOCAN Estimates of Incidence and Mortality Worldwide for 36 Cancers in 185 Countries. CA Cancer J. Clin. 68 (6), 394–424. 10.3322/caac.21492 30207593

[B8] Briggs A. S. M. Claxton K. (2006). Decision Modelling for Health Economic Evaluation. UK: Oxford University Press.

[B9] Centers for Medicare and Medicaid Services (2021a). ASP Drug Pricing Files. Availableat: https://www.cms.gov/medicare/medicare-part-b-drug-average-sales-price/2021-asp-drug-pricing-files (Accessed July 24,2021)

[B10] Centers for Medicare and Medicaid Services (2021b). Medicare Physician Fee Schedule Look-Up Tool. Availableat: https://www.cms.gov/medicare/physician-fee-schedule/search (Accessed January 2, 2021)

[B11] Creelan B. C. (2014). Update on Immune Checkpoint Inhibitors in Lung Cancer. Cancer Control 21 (1), 80–89. 10.1177/107327481402100112 24357746

[B12] Criss S. D. Mooradian M. J. Watson T. R. Gainor J. F. Reynolds K. L. Kong C. Y. (2019). Cost-effectiveness of Atezolizumab Combination Therapy for First-Line Treatment of Metastatic Nonsquamous Non-small Cell Lung Cancer in the United States. JAMA Netw. Open 2 (9), e1911952. 10.1001/jamanetworkopen.2019.11952 31553470 PMC6764123

[B13] Comparative price Report (2013). Variation in Medical and Hospital Prices by Country. International Federation of Health Plans. Availableat: http://www.ifhp.com/1404121. (Accessed May 15, 2021).

[B14] D'Incecco A. Andreozzi M. Ludovini V. Rossi E. Capodanno A. Landi L. (2015). PD-1 and PD-L1 Expression in Molecularly Selected Non-small-cell Lung Cancer Patients. Br. J. Cancer 112 (1), 95–102. 10.1038/bjc.2014.555 25349974 PMC4453606

[B15] Di Federico A. De Giglio A. Parisi C. Gelsomino F. Ardizzoni A. (2021). PD-1/PD-L1 Inhibitor Monotherapy or in Combination with Chemotherapy as Upfront Treatment for Advanced NSCLC with PD-L1 Expression ≥ 50%: Selecting the Best Strategy. Crit. Rev. Oncol. Hematol. 160, 103302. 10.1016/j.critrevonc.2021.103302 33753247

[B16] Duckett S. J. (2004). Drug Policy Down under: Australia's Pharmaceutical Benefits Scheme. Health Care Financ. Rev. 25 (3), 55–67. 15229996 PMC4194861

[B17] Ettinger D. S. Wood D. E. Aisner D. L. Akerley W. Bauman J. R. Bharat A. (2021). NCCN Guidelines Insights: Non-small Cell Lung Cancer, Version 2.2021. J. Natl. Compr. Canc Netw. 19 (3), 254–266. 10.6004/jnccn.2021.0013 33668021

[B18] Gridelli C. Casaluce F. (2018). Frontline Immunotherapy for NSCLC: Alone or Not Alone? Nat. Rev. Clin. Oncol. 15 (10), 593–594. 10.1038/s41571-018-0070-7 29993034

[B19] Hanna N. H. Schneider B. J. Temin S. Baker S. Jr Brahmer J. Ellis P. M. (2020). Therapy for Stage IV Non-small-cell Lung Cancer without Driver Alterations: ASCO and OH (CCO) Joint Guideline Update. J. Clin. Oncol. 38 (14), 1608–1632. 10.1200/JCO.19.03022 31990617

[B20] Hatswell A. J. Pennington B. Pericleous L. Rowen D. Lebmeier M. Lee D. (2014). Patient-reported Utilities in Advanced or Metastatic Melanoma, Including Analysis of Utilities by Time to Death. Health Qual. Life Outcomes 12, 140. 10.1186/s12955-014-0140-1 25214238 PMC4173059

[B21] Herbst R. S. Giaccone G. de Marinis F. Reinmuth N. Vergnenegre A. Barrios C. H. (2020). Atezolizumab for First-Line Treatment of PD-L1-Selected Patients with NSCLC. N. Engl. J. Med. 383 (14), 1328–1339. 10.1056/NEJMoa1917346 32997907

[B22] Huang M. Lou Y. Pellissier J. Burke T. Liu F. X. Xu R. (2017). Cost Effectiveness of Pembrolizumab vs. Standard-Of-Care Chemotherapy as First-Line Treatment for Metastatic NSCLC that Expresses High Levels of PD-L1 in the United States. Pharmacoeconomics 35 (8), 831–844. 10.1007/s40273-017-0527-z 28620848 PMC5548835

[B23] Kerr K. M. Tsao M. S. Nicholson A. G. Yatabe Y. Wistuba II Hirsch F. R. IASLC Pathology Committee (2015). Programmed Death-Ligand 1 Immunohistochemistry in Lung Cancer: In what State Is This Art? J. Thorac. Oncol. 10 (7), 985–989. 10.1097/JTO.0000000000000526 26134220

[B24] Kim R. Keam B. Hahn S. Ock C. Y. Kim M. Kim T. M. (2019). First-line Pembrolizumab versus Pembrolizumab Plus Chemotherapy versus Chemotherapy Alone in Non-small-cell Lung Cancer: A Systematic Review and Network Meta-Analysis. Clin. Lung Cancer 20 (5), 331–e4. 10.1016/j.cllc.2019.05.009 31164319

[B25] Mailankody S. Prasad V. (2014). Comparative Effectiveness Questions in Oncology. N. Engl. J. Med. 370 (16), 1478–1481. 10.1056/NEJMp1400104 24738667

[B26] Mailankody S. Prasad V. (2015). Five Years of Cancer Drug Approvals: Innovation, Efficacy, and Costs. JAMA Oncol. 1 (4), 539–540. 10.1001/jamaoncol.2015.0373 26181265

[B27] Miller K. D. Fidler-Benaoudia M. Keegan T. H. Hipp H. S. Jemal A. Siegel R. L. (2020). Cancer Statistics for Adolescents and Young Adults, 2020. CA Cancer J. Clin. 70 (1), 443–459. 10.3322/caac.2165410.3322/caac.21637 32940362

[B28] Mok T. S. K. Wu Y. L. Kudaba I. Kowalski D. M. Cho B. C. Turna H. Z. (2019). Pembrolizumab versus Chemotherapy for Previously Untreated, PD-L1-Expressing, Locally Advanced or Metastatic Non-small-cell Lung Cancer (KEYNOTE-042): a Randomised, Open-Label, Controlled, Phase 3 Trial. Lancet 393 (10183), 1819–1830. 10.1016/S0140-6736(18)32409-7 30955977

[B29] Nafees B. Lloyd A. J. Dewilde S. Rajan N. Lorenzo M. (2017). Health State Utilities in Non-small Cell Lung Cancer: An International Study. Asia Pac. J. Clin. Oncol. 13 (5), e195–e203. 10.1111/ajco.12477 26990789

[B30] National Cancer Institute Surveillance E., and End Results Program (2021). SEER*Stat Software Version 8.3.9. Availableat: https://seer.cancer.gov/seerstat/software/ . (Accessed on January 8, 2021)

[B31] Neumann P. J. Cohen J. T. Weinstein M. C. (2014). Updating Cost-Effectiveness-Tthe Curious Resilience of the $50,000-Per-QALY Threshold. N. Engl. J. Med. 371 (9), 796–797. 10.1056/NEJMp1405158 25162885

[B32] Peng Y. Zeng X. Peng L. Liu Q. Yi L. Luo X. (2021). First-Line Atezolizumab for Metastatic NSCLC with High PD-L1 Expression: A United States-Based Cost-Effectiveness Analysis. Adv. Ther. 38 (5), 2447–2457. 10.1007/s12325-021-01734-6 33821431

[B33] Prasad V. Mailankody S. (2016). The UK Cancer Drugs Fund Experiment and the US Cancer Drug Cost Problem: Bearing the Cost of Cancer Drugs until it Is Unbearable. Mayo Clin. Proc. 91 (6), 707–712. 10.1016/j.mayocp.2016.04.028 27261866

[B34] Reck M. Rodríguez-Abreu D. Robinson A. G. Hui R. Csőszi T. Fülöp A. (2016). Pembrolizumab versus Chemotherapy for PD-L1-Positive Non-small-cell Lung Cancer. N. Engl. J. Med. 375 (19), 1823–1833. 10.1056/NEJMoa1606774 27718847

[B35] Sezer A. Kilickap S. Gümüş M. Bondarenko I. Özgüroğlu M. Gogishvili M. (2021). Cemiplimab Monotherapy for First-Line Treatment of Advanced Non-small-cell Lung Cancer with PD-L1 of at Least 50%: a Multicentre, Open-Label, Global, Phase 3, Randomised, Controlled Trial. Lancet 397 (10274), 592–604. 10.1016/S0140-6736(21)00228-2 33581821

[B36] Tang M. Song P. He J. (2020). Progress on Drug Pricing Negotiations in China. Biosci. Trends 13 (6), 464–468. 10.5582/bst.2019.01339 31875587

[B37] Trusheim M. R. Cassidy W. M. Bach P. B. (2018). Alternative State-Level Financing for Hepatitis C Treatment-The "Netflix Model". JAMA 320 (19), 1977–1978. 10.1001/jama.2018.15782 30383176

[B38] Tsevat J. Moriates C. (2018). Value-Based Health Care Meets Cost-Effectiveness Analysis. Ann. Intern. Med. 169 (5), 329–332. 10.7326/M18-0342 30083766

[B39] Us Food and Drug Administration (2021a). FDA Approves Atezolizumab for First-Line Treatment of Metastatic NSCLC with High PD-L1 Expression. Availableat: https://www.fda.gov/drugs/resources-information-approved-drugs/fda-approves-atezolizumab-first-line-treatment-metastatic-nsclc-high-pd-l1-expression . (Accessed March 5, 2021)

[B40] Us Food and Drug Administration (2021b). FDA Approves Cemiplimab-Rwlc for Non-small Cell Lung Cancer with High PD-L1 Expression. Availableat: https://www.fda.gov/drugs/resources-information-approved-drugs/fda-approves-cemiplimab-rwlc-non-small-cell-lung-cancer-high-pd-l1-expression (Accessed May 2, 2021)

[B41] Us Food and Drug Administration (2016). Pembrolizumab (KEYTRUDA) Checkpoint Inhibitor. Availableat: https://www.fda.gov/drugs/resources-information-approved-drugs/pembrolizumab-keytruda-checkpoint-inhibitor .(Accessed June 26, 2021)

[B42] Wan X. Zhang Y. Tan C. Zeng X. Peng L. (2019). First-line Nivolumab Plus Ipilimumab vs Sunitinib for Metastatic Renal Cell Carcinoma: A Cost-Effectiveness Analysis. JAMA Oncol. 5 (4), 491–496. 10.1001/jamaoncol.2018.7086 30789633 PMC6459127

[B43] Watson T. R. Gao X. Reynolds K. L. Kong C. Y. (2020). Cost-effectiveness of Pembrolizumab Plus Axitinib vs Nivolumab Plus Ipilimumab as First-Line Treatment of Advanced Renal Cell Carcinoma in the US. JAMA Netw. Open 3 (10), e2016144. 10.1001/jamanetworkopen.2020.16144 33052401 PMC7557509

[B44] William W. N. Jr. Lin H. Y. Lee J. J. Lippman S. M. Roth J. A. Kim E. S. (2009). Revisiting Stage IIIB and IV Non-small Cell Lung Cancer: Analysis of the Surveillance, Epidemiology, and End Results Data. Chest 136 (3), 701–709. 10.1378/chest.08-2968 19318668

[B45] Wu B. Dong B. Xu Y. Zhang Q. Shen J. Chen H. (2012). Economic Evaluation of First-Line Treatments for Metastatic Renal Cell Carcinoma: a Cost-Effectiveness Analysis in a Health Resource-Limited Setting. PloS one 7 (3), e32530. 10.1371/journal.pone.0032530 22412884 PMC3297611

[B46] Zhang L. Hang Y. Liu M. Li N. Cai H. (2020). First-Line Durvalumab Plus Platinum-Etoposide versus Platinum-Etoposide for Extensive-Stage Small-Cell Lung Cancer: A Cost-Effectiveness Analysis. Front. Oncol. 10, 602185. 10.3389/fonc.2020.602185 33344252 PMC7747765

