# Supplementary figures and images for: First-Line ICI Monotherapies for Advanced Non-small-cell Lung Cancer Patients With PD-L1 of at Least 50%: A Cost-Effectiveness Analysis
Source: Front Pharmacol. 2021 Dec 21;12:788569. doi: 10.3389/fphar.2021.788569 (PMC8724566; doi:10.3389/fphar.2021.788569)

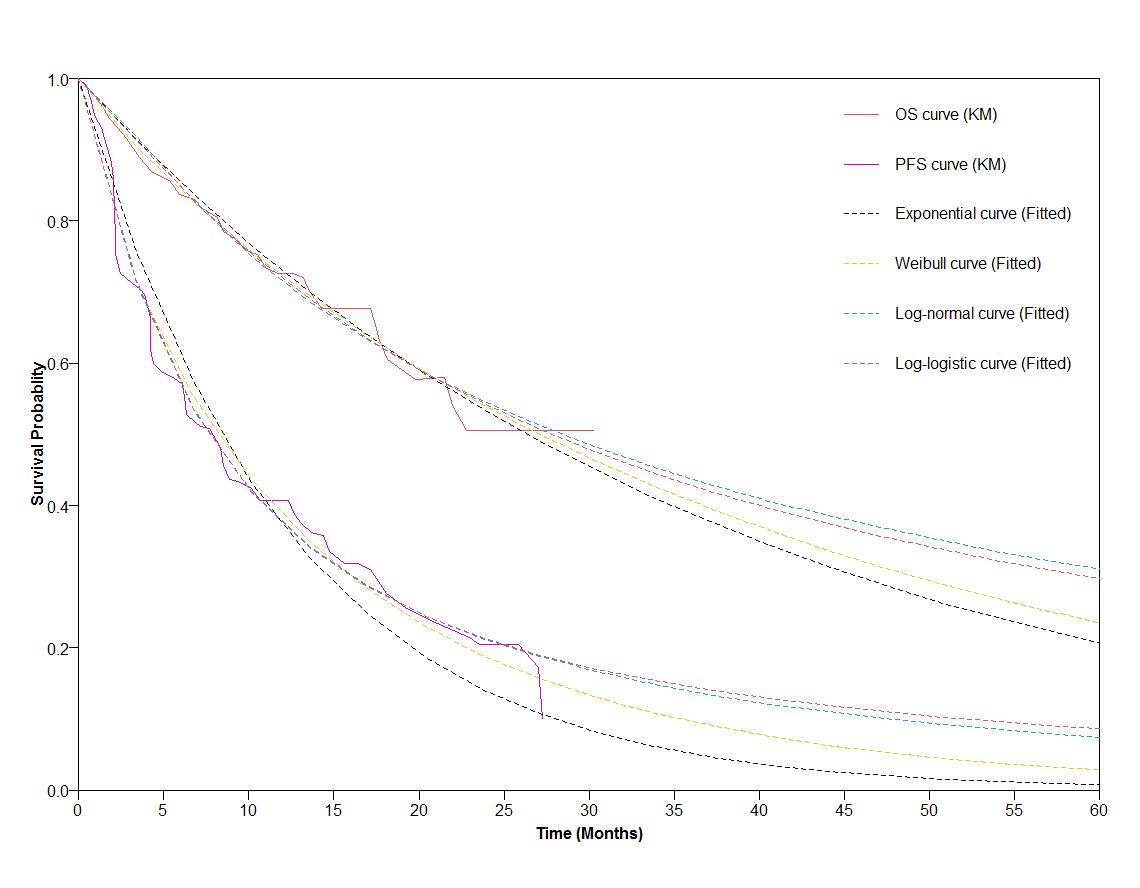

Supplement: Supplementary file 1 [file Image1.TIFF]

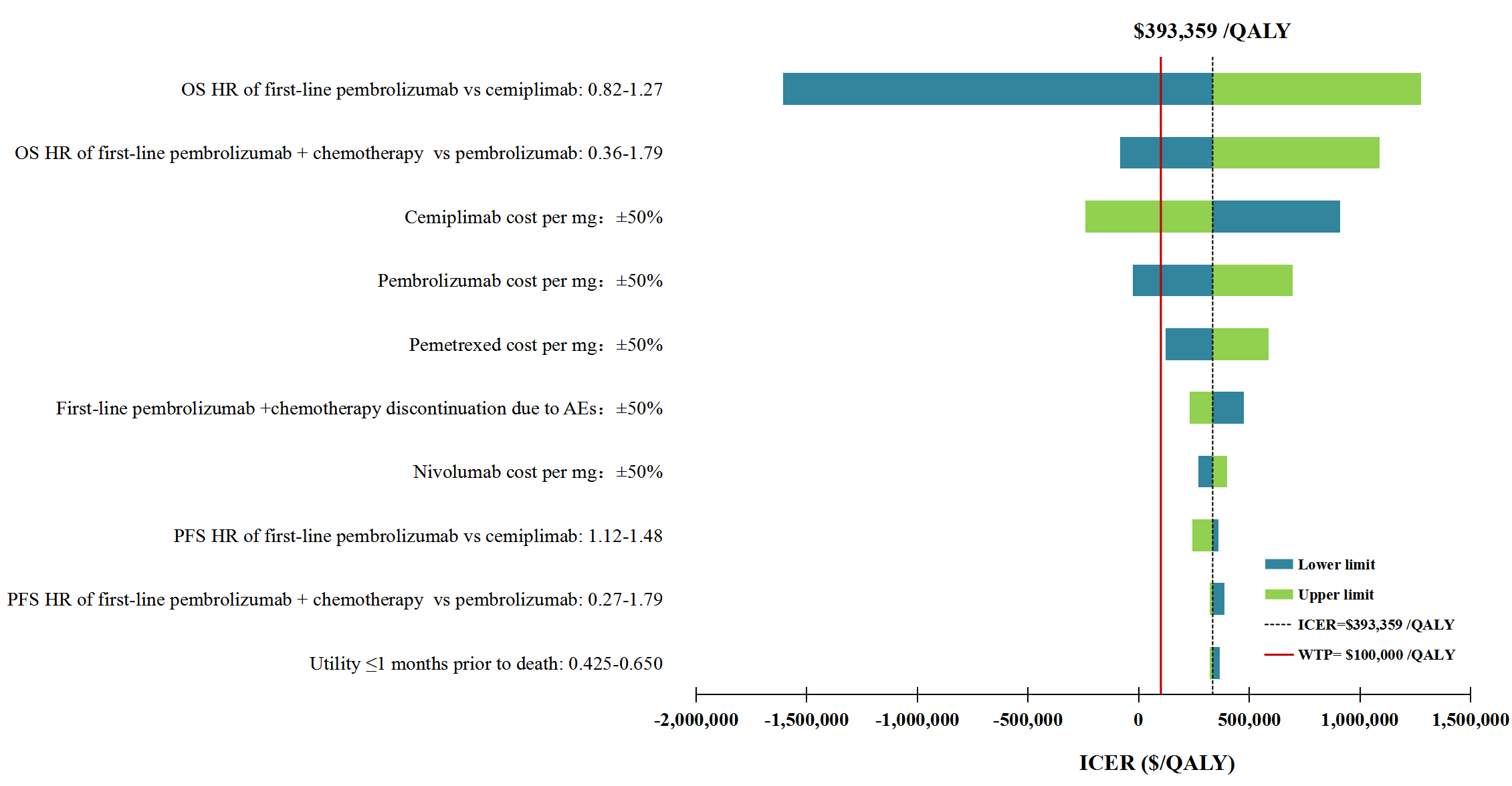

Supplement: Supplementary file 4 [file Image3.TIF]

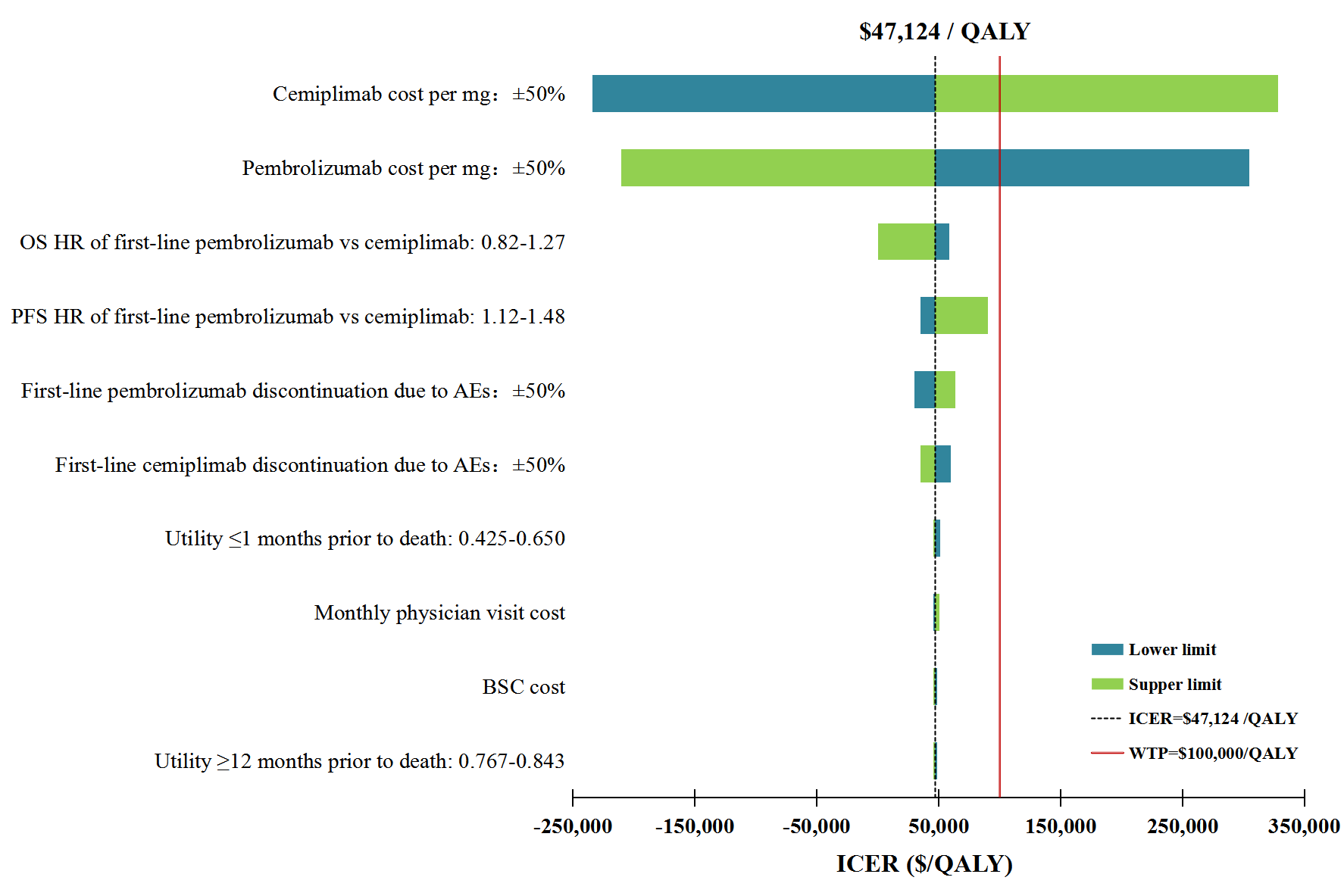

Supplement: Supplementary file 6 [file Image2.TIF]
