# Supplementary material for: First-Line ICI Monotherapies for Advanced Non-small-cell Lung Cancer Patients With PD-L1 of at Least 50%: A Cost-Effectiveness Analysis
Source: Front Pharmacol. 2021 Dec 21;12:788569. doi: 10.3389/fphar.2021.788569 (PMC8724566; doi:10.3389/fphar.2021.788569)
Supplement: Supplementary file 2 [file DataSheet4.docx]

Table 4. Proportion and probability of first-line treatment discontinuation due to AEs.

| **Regimen** | **Proportion** | **Instantaneous rate** | **1-cyle probabilities** |
| --- | --- | --- | --- |
| First-line cemiplimab | 0.06479 | 0.00212 | 0.00212 |
| First-line pembrolizumab | 0.12338 | 0.00350 | 0.00350 |
| First-line atezolizumab | 0.03738 | 0.00132 | 0.00132 |

*AEs, adverse events.*
