# Supplementary material for: First-Line ICI Monotherapies for Advanced Non-small-cell Lung Cancer Patients With PD-L1 of at Least 50%: A Cost-Effectiveness Analysis
Source: Front Pharmacol. 2021 Dec 21;12:788569. doi: 10.3389/fphar.2021.788569 (PMC8724566; doi:10.3389/fphar.2021.788569)
Supplement: Supplementary file 3 [file DataSheet6.docx]

Table 6. Proportion, costs and disutility of grade III/IV AEs considered in the model.

| **AEs** | **Proportion (%)** | | | **Cost per event ($)** | **Disutility** |
| --- | --- | --- | --- | --- | --- |
|  | **Cemiplimab^1^** | **Pembrolizumab** | **Atezolizumab** |  |  |
| Anemia | 0.00% | 1.95% | 1.75% | 7905 | /^b^ |
| Increased aspartate aminotransferase | 1.41% | 0.00% | 0.00% | 16488 | /^b^ |
| Pneumonia | 1.13% | 2.60% | 2.45% | 10450 | /^b^ |
| Severe skin reaction | 0.00% | 3.90% | 0.00% | 10606 | /^b^ |
| Colitis | 0.00% | 1.30% | 0.00% | 12118 | /^b^ |
| Constipation | 0.00% | 0.00% | 1.05% | 13378 | /^b^ |
| Hyponatremia | 0.00% | 0.00% | 2.10% | 7624 | /^b^ |
| Hyperkalemia | 0.00% | 0.00% | 2.10% | 7624 | /^b^ |
| Neutropenia | 0.56% | 0.00% | 0.70% | 14319 | 0.35 |
| Fatigue | 0.85% | 1.30% | 0.70% | 11231 | 0.29 |
| Diarrhoea | 0.28% | 3.90% | 0.00% | 13378 | 0.22 |
| Vomiting | 0.00% | 0.65% | 0.35% | Not considered in the model^a^ | 0.20 |
| Rash | 0.85% | 0.00% | 0.00% | Not considered in the model^a^ | 0.15 |
| **Estimated AEs Costs and disutility** | | | |  |  |
| AEs cost for cemiplimab group, $ | | | | 563.24 |  |
| AEs cost for pembrolizumab group, $ | | | | 1663.10 |  |
| AEs cost for atezolizumab group, $ | | | | 1032.86 |  |
| AEs disutility for cemiplimab group | | | |  | 0.006 |
| AEs disutility for pembrolizumab group | | | |  | 0.014 |
| AEs disutility for atezolizumab group | | | |  | 0.005 |

*AEs, adverse events.*

*^a^The cost of grade III/IV AEs with an incidence of at least 1% in any group were incorporated the model.*

*^b^The utility decrements regarding those AEs were not reported.*
