# Supplementary material for: First-Line ICI Monotherapies for Advanced Non-small-cell Lung Cancer Patients With PD-L1 of at Least 50%: A Cost-Effectiveness Analysis
Source: Front Pharmacol. 2021 Dec 21;12:788569. doi: 10.3389/fphar.2021.788569 (PMC8724566; doi:10.3389/fphar.2021.788569)
Supplement: Supplementary file 5 [file DataSheet3.docx]

Table 3. Studies included in the network meta-analysis.

| **Study** | **Experimental Arm** | **Control Arm** | **OS HR**  **(95% CI)** | **PFS HR (95% CI)** |
| --- | --- | --- | --- | --- |
| EMPOWER-Lung 1^*^ | Cemiplimab  (n = 283) | Platinum-based chemotherapy  (n = 280) | 0.57  (0.42-0.70) | 0.54  (0.43-0.68) |
| KEYNOTE-024^*^ | Pembrolizumab  (n = 154) | Platinum-based  chemotherapy  (n = 151) | 0.62（0.41-0.89） | 0.50  (0.37-0.68） |
| KEYNOTE-042^**^ | Pembrolizumab  (n = 299) | Platinum-based  chemotherapy  (n = 300) | 0.69（0.56-0.85） | 0.81  (0.67-0.99) |
| IMpower110^**^ | Atezolizumab  (n = 107) | Platinum-based  chemotherapy  (n = 98) | 0.59 (0.40-0.89) | 0.63 (0.45-0.88) |

*^*^The EMPOWER-Lung 1 and the KEYNOTE-024 trials only enrolled patients with a PD-L1 tumor proportion score of 50% or greater. ^**^ The IMpower-110 and the KEYNOTE-042 studies, patients had a PD-L1 tumor proportion score of 1% or greater were included. In this network meta-analysis, in order to compare homogenous populations, only subjects with PD-L1≥50% were considered.*
