# Supplementary material for: First-Line ICI Monotherapies for Advanced Non-small-cell Lung Cancer Patients With PD-L1 of at Least 50%: A Cost-Effectiveness Analysis
Source: Front Pharmacol. 2021 Dec 21;12:788569. doi: 10.3389/fphar.2021.788569 (PMC8724566; doi:10.3389/fphar.2021.788569)
Supplement: Supplementary file 7 [file DataSheet5.docx]

Table 5. SEER survival statistic: advanced NSCLC (2000-2018).

| **Months** | **Survival estimates** | **Months** | **Survival estimates** | **Months** | **Survival estimates** | **Months** | **Survival estimates** |
| --- | --- | --- | --- | --- | --- | --- | --- |
| 25 | 9.40% | 49 | 3.87% | 73 | 2.39% | 97 | 1.71% |
| 26 | 8.84% | 50 | 3.78% | 74 | 2.35% | 98 | 1.70% |
| 27 | 8.43% | 51 | 3.65% | 75 | 2.33% | 99 | 1.67% |
| 28 | 8.01% | 52 | 3.58% | 76 | 2.30% | 100 | 1.66% |
| 29 | 7.66% | 53 | 3.52% | 77 | 2.27% | 101 | 1.65% |
| 30 | 7.35% | 54 | 3.43% | 78 | 2.21% | 102 | 1.62% |
| 31 | 7.02% | 55 | 3.37% | 79 | 2.18% | 103 | 1.59% |
| 32 | 6.76% | 56 | 3.28% | 80 | 2.15% | 104 | 1.56% |
| 33 | 6.47% | 57 | 3.21% | 81 | 2.11% | 105 | 1.55% |
| 34 | 6.25% | 58 | 3.15% | 82 | 2.07% | 106 | 1.54% |
| 35 | 6.00% | 59 | 3.11% | 83 | 2.04% | 107 | 1.53% |
| 36 | 5.80% | 60 | 3.03% | 84 | 2.03% | 108 | 1.50% |
| 37 | 5.59% | 61 | 2.99% | 85 | 2.00% | 109 | 1.48% |
| 38 | 5.42% | 62 | 2.94% | 86 | 1.98% | 110 | 1.47% |
| 39 | 5.24% | 63 | 2.89% | 87 | 1.97% | 111 | 1.45% |
| 40 | 5.09% | 64 | 2.84% | 88 | 1.94% | 112 | 1.43% |
| 41 | 4.93% | 65 | 2.80% | 89 | 1.92% | 113 | 1.42% |
| 42 | 4.77% | 66 | 2.73% | 90 | 1.90% | 114 | 1.42% |
| 43 | 4.61% | 67 | 2.68% | 91 | 1.87% | 115 | 1.40% |
| 44 | 4.49% | 68 | 2.63% | 92 | 1.84% | 116 | 1.37% |
| 45 | 4.38% | 69 | 2.56% | 93 | 1.81% | 117 | 1.35% |
| 46 | 4.23% | 70 | 2.52% | 94 | 1.78% | 118 | 1.34% |
| 47 | 4.09% | 71 | 2.49% | 95 | 1.76% | 119 | 1.33% |
| 48 | 3.98% | 72 | 2.44% | 96 | 1.74% | 120 | 1.31% |

*SEER, Surveillance, Epidemiology, and End Results; NSCLC, Non-small cell lung cancer.*
