# Supplementary material for: First-Line ICI Monotherapies for Advanced Non-small-cell Lung Cancer Patients With PD-L1 of at Least 50%: A Cost-Effectiveness Analysis
Source: Front Pharmacol. 2021 Dec 21;12:788569. doi: 10.3389/fphar.2021.788569 (PMC8724566; doi:10.3389/fphar.2021.788569)
Supplement: Supplementary file 8 [file DataSheet2.docx]

Table 2. Parametric survival distributions fitted for first-line cemiplimab.

| **Parametric Model** | **OS data** | | **PFS data** | |
| --- | --- | --- | --- | --- |
|  | **AIC** | **BIC** | **AIC** | **BIC** |
| Exponential | -199.52 | -196.09 | -161.72 | -157.78 |
| Weibull | -141.32 | -136.83 | -196.52 | -190.61 |
| Lognormal | -125.74 | -186.83 | -223.57 | -217.66 |
| Loglogistic | -199.72 | -199.58 | -235.72 | -239.81 |

*OS, overall survival; PFS, progression-free survival; AIC, Akaike information criterion; BIC, Bayesian information criterion.*
