# Supplementary material for: First-Line ICI Monotherapies for Advanced Non-small-cell Lung Cancer Patients With PD-L1 of at Least 50%: A Cost-Effectiveness Analysis
Source: Front Pharmacol. 2021 Dec 21;12:788569. doi: 10.3389/fphar.2021.788569 (PMC8724566; doi:10.3389/fphar.2021.788569)
Supplement: Supplementary file 9 [file DataSheet1.docx]

Table 1. First-line and subsequent treatment regimes in the model.

| **Regimen** | **Dosage and Schedule** | **Median exposure duration** | **Subsequent anti-cancer therapy** | | |
| --- | --- | --- | --- | --- | --- |
|  |  |  | **Regimen** | **Dosage and Schedule** | **proportion** |
| First-line  Cemiplimab | 350mg every 3-week cycles up to 36 cycles | 9 cycles | Cemiplimab+ Docetaxel | Cemiplimab, 350mg every 3-week cycle up to additional 36 cycles;  docetaxel,75 mg/m2 for 4 cycles; | 31.6% |
| First-line  Pembrolizumab | 200mg every 3-week cycles up to 35 cycles | 10 cycles | Docetaxel | docetaxel,75 mg/m^2^ for 4 cycles | 46.6% |
|  |  |  | Ramucirumab | 15mg/kg every 3-week cycle | 7.5% |
|  |  |  | Nivolumab | 3mg/kg every 2-week | 3.0% |
|  |  |  | Pembrolizumab | 200mg every 3-week cycle | 0.8% |
| First-line  Atezolizumab | 1200mg every 3-week cycles | 8 cycles | Docetaxel | docetaxel,75 mg/m^2^ for 4 cycles | 6.6% |
|  |  |  | Pemetrexed+ platinum | Pemetrexed,500 mg/m^2^ every 3-week cycle;  cisplatin,75 mg/m^2^ or carboplatin, AUC 5.0 mg/ml/min for 4 cycles | 10.5% |
|  |  |  | Gemcitabine+ platinum | Gemcitabine,1250 mg/m^2^ for 4 cycles;  cisplatin,75 mg/m^2^ or carboplatin, AUC 5.0 mg/ml/min for 4 cycles | 10.5% |
|  |  |  | Paclitaxel + platinum | Paclitaxel, 200 mg/m^2^ for 4 cycles;  cisplatin,75 mg/m^2^ or carboplatin, AUC 6.0 mg/ml/min for 4 cycles | 9.2% |
|  |  |  | Ramucirumab | 15mg/kg every 3-week cycle | 2.6% |
|  |  |  | Nivolumab | 4.5 mg/kg every 3-week cycle | 11.8% |
